# Characterization of Genipin-Modified Dentin Collagen

**DOI:** 10.1155/2014/702821

**Published:** 2014-03-25

**Authors:** Hiroko Nagaoka, Hideaki Nagaoka, Ricardo Walter, Lee W. Boushell, Patricia A. Miguez, Andrew Burton, André V. Ritter, Mitsuo Yamauchi

**Affiliations:** ^1^Department of Operative Dentistry, School of Dentistry, University of North Carolina at Chapel Hill, Chapel Hill, NC 27599, USA; ^2^NC Oral Health Institute, School of Dentistry, University of North Carolina at Chapel Hill, Chapel Hill, NC 27599, USA; ^3^Department of Preventive and Restorative Sciences, School of Dental Medicine, University of Pennsylvania, Philadelphia, PA 19104, USA; ^4^Department of Periodontics, School of Dental Medicine, University of Pennsylvania, Philadelphia, PA 19104, USA

## Abstract

Application of biomodification techniques to dentin can improve its biochemical and biomechanical properties. Several collagen cross-linking agents have been reported to strengthen the mechanical properties of dentin. However, the characteristics of collagen that has undergone agent-induced biomodification are not well understood. The objective of this study was to analyze the effects of a natural cross-linking agent, genipin (GE), on dentin discoloration, collagen stability, and changes in amino acid composition and lysyl oxidase mediated natural collagen cross-links. Dentin collagen obtained from extracted bovine teeth was treated with three different concentrations of GE (0.01%, 0.1%, and 0.5%) for several treatment times (0–24 h). Changes in biochemical properties of NaB^3^H_4_-reduced collagen were characterized by amino acid and cross-link analyses. The treatment of dentin collagen with GE resulted in a concentration- and time-dependent pigmentation and stability against bacterial collagenase. The lysyl oxidase-mediated trivalent mature cross-link, pyridinoline, showed no difference among all groups while the major divalent immature cross-link, dehydro-dihydroxylysinonorleucine/its ketoamine in collagen treated with 0.5% GE for 24 h, significantly decreased compared to control (*P* < 0.05). The newly formed GE-induced cross-links most likely involve lysine and hydroxylysine residues of collagen in a concentration-dependent manner. Some of these cross-links appear to be reducible and stabilized with NaB^3^H_4_.

## 1. Introduction

Fibrillar type I collagen is the major organic component in dentin matrix and functions as a stable template to spatially regulate mineral deposition and growth [1–3]. One of the functionally important characteristics of collagen is its unique posttranslational modifications. Covalent intermolecular cross-linking is the final posttranslational modification and is crucial for the stability, tensile strength, and viscoelasticity of collagen matrix [4–8].

Lysyl oxidase (LOX-) mediated collagen cross-linking has been extensively studied [5, 9–12]. The chemical structure and the quantity of these cross-links are primarily determined by the extent of hydroxylation of specific lysine (Lys) residues in the collagen molecule and the extent of oxidative deamination of the Lys and hydroxylysine (Hyl) residues in the telopeptide domains of the molecule. Lysyl hydroxylases and LOX/LOX-like proteins catalyze the reactions for these modifications, respectively. The glycosylation pattern of specific helical Hyl residues that are involved in cross-linking may modulate the maturation of collagen cross-links [13]. The cross-linking pattern can also be determined by the maturation/turnover rate of tissues [14–18], the details of molecular packing structure [18–20], and the physical force exerted on the tissue [21].

Collagen cross-links can also be induced nonenzymatically by treatment with chemicals and natural plant/fruit extracts [22]. Such cross-linking has been shown to facilitate preservation of substrate shape in scaffolds [23], improve physiological function of tissues [24–27], and increase the mechanical properties of collagen [22, 28–30]. For instance, the synthetic cross-linking agent glutaraldehyde (GA) has been widely used as a fixative agent. It is well documented that GA treatment improves the stability of various collagen-based tissues [26, 31–33]. However, the direct application of GA to biological tissues has limitation due to its cytotoxicity [24, 34].

Genipin (GE), a traditional Chinese herbal medicine extracted from fruit of* Gardenia jasminoides,* has been found to be an effective collagen cross-linking agent [35]. GE has relatively mild* in vitro* cytotoxicity, and GE-treated collagen has increased toughness when compared to GA-treated collagen [24, 36].

While several studies on the effects of GE on dentin have been published [30, 37, 38], experimental conditions for GE treatment are not well defined and the nature of GE-induced cross-linking remains elusive. A better understanding of GE-induced collagen modification in dentin may provide insights into the development of novel biomodification technology which is useful in dentistry. As the first step towards this goal, we characterized the effects of various GE treatment regimens on dentin discoloration, collagen stability, amino acid composition, and LOX-mediated collagen cross-links.

## 2. Materials and Methods

### 2.1. Sample Collection, Demineralization, and GE Treatment

Extracted intact bovine incisors (≤1 year old animals) were used in this study. Enamel, cementum, and pulp were removed using high-speed diamond burs with water/air-cooling. Dentin was pulverized under liquid N_2_ by a Spex Freezer Mill (SPEX CertiPrep, Inc., Metuchen, NJ, USA) and extensively washed with cold distilled water and lyophilized. The resultant dentin powder was demineralized with 0.5 M ethylenediaminetetraacetic acid (EDTA, pH 7.4) at 4°C for 14 days. The EDTA solution was changed twice each week during the demineralization period. The demineralized dentin matrix protein (~90% collagen) was extensively washed with cold distilled water and lyophilized. 2 mg aliquots of the collagen were randomly allocated to the following four treatment groups based on GE concentration (*n* = 21/group).

 Group 1 (control): specimens were treated with phosphate buffered saline (PBS).
 Group 2 (0.01% GE): specimens were treated with 0.01% GE in PBS.
 Group 3 (0.1% GE): specimens were treated with 0.1% GE in PBS.
 Group 4 (0.5% GE): specimens were treated with 0.5% GE in PBS.

Previously, ~0.5% of GE treatment has been shown to improve dentin mechanical properties [30, 38] but lower concentrations have not been investigated in this context.

### 2.2. Discoloration and Stability of GE-Modified Dentin Collagen

2 mg aliquots of demineralized dentin collagen were treated with 1 mL of PBS or GE (Wako Pure Chemical Industries, Ltd., Osaka, Japan) according to groups 1–4 and incubated for 30 min, 1 h, 4 h, 8 h, 12 h, and 24 h at 37°C with agitation (*n* = 3/time point, *n* = 18/group). After treatment, the samples were extensively washed with distilled water, lyophilized, and observed for their discoloration.

Collagen stability was assessed in the same samples by enzymatic degradation assay as reported [30]. Samples were suspended in 1 mL of 50 mM ammonium bicarbonate and digested with 5% w/w bacterial collagenase derived from* Clostridium histolyticum *(Worthington Biochemical Corp., Lakewood, NJ, USA, 1,075 U/mg) for 24 h at 37°C. After digestion, the samples were centrifuged for 15 min at 15,000 g, and the residues (undigested collagen) were washedwith distilled water by repeated centrifugation and lyophilized. The residues were hydrolyzed with 300 *μ*L of 6 N HCl (Pierce, Rockford, IL, USA)* in vacuo* after flushing with N_2_ gas for 22 h at 105°C. The hydrolysates were dried, reconstituted in 300 *μ*L of distilled water, filtered, and subjected to hydroxyproline (Hyp) analysis using a high-performance liquid chromatography (HPLC) system (Prostar 240/310, Varian, Walnut Creek, CA, USA) fitted with a cation exchange column (AA911; Transgenomic, Inc., San Jose, CA, USA) [39]. The primary outcome measure for this analysis was the Hyp recovered in the undigested residue in nM, which is expressed as means ± standard deviation (SD).

### 2.3. Amino Acid and Collagen Cross-Link Analyses

2 mg aliquots of demineralized dentin collagen were treated with PBS or GE according to groups 1–4 and incubated for 24 h at 37°C (*n* = 3/group). After treatment, the samples were extensively washed with distilled water, lyophilized, reduced with standardized NaB^3^H_4_ to stabilize and label reducible cross-links, and hydrolyzed. An aliquot of each hydrolysate was subjected to amino acid analysis as described [39]. The relative amount of each amino acid was calculated as residues per 1,000 total amino acids. The hydrolysates with known amounts of Hyp were then analyzed for cross-links [39]. The major reducible immature cross-links, dehydrodihydroxylysinonorleucine (deH-DHLNL)/its ketoamine and dehydrohydroxylysinonorleucine/its ketoamine (deH-HLNL), were analyzed as their reduced forms, that is, dihydroxylysinonorleucine (DHLNL) and hydroxylysinonorleucine (HLNL), respectively. The nonreducible mature cross-links, pyridinoline (Pyr) and deoxy-Pyr (d-Pyr), were also analyzed simultaneously as described [39]. For their chemical structures, see [5]. The major cross-links in dentin collagen, DHLNL and Pyr, were quantified as moles/mole of collagen based on the value of 300 residues of Hyp per collagen molecule. All analyses were done in triplicate in independent experiments.

### 2.4. Statistical Analysis

The statistical analyses were performed using two-way ANOVA and Fisher's PLSD (Stat View software, SAS Institute Inc., Cary, NC,USA). A *P* value of less than 0.05 was considered statistically significant.

## 3. Results

### 3.1. Discoloration and Stability of GE-Modified Dentin Collagen

**Figure 1:**
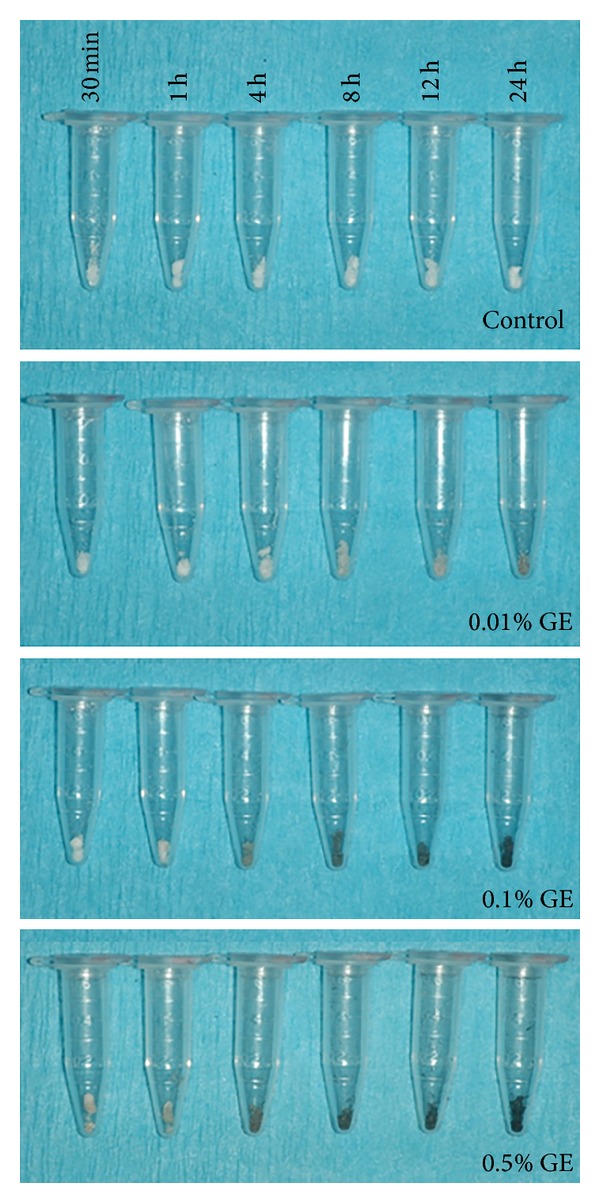
Discoloration of GE-modified dentin collagen. Photographs of the representative discoloration of dentin collagen treated with PBS (control) and three different concentrations (0.01%, 0.1%, and 0.5%) of genipin (GE) for six treatment durations (30 min, 1 h, 4 h, 8 h, 12 h, and 24 h).

**Figure 2:**
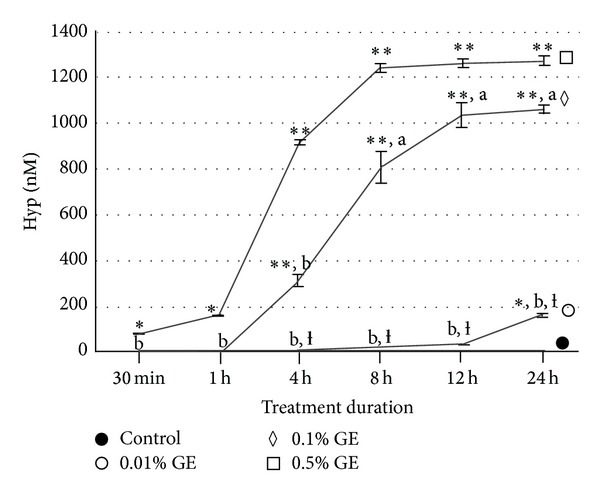
Collagen stability of GE-modified dentin collagen. 2 mg aliquots of demineralized dentin treated with PBS (control) and three concentrations of genipin (GE) (0.01%, 0.1%, and 0.5%) for six treatment durations (30 min, 1 h, 4 h, 8 h, 12 h, and 24 h) were subjected to collagenase digestion and hydroxyproline (Hyp) analysis. The mean amounts of undigested collagen with bacterial collagenase are shown as Hyp content (nM) in the insoluble residues. (*n* = 3). *P < 0.001, **P < 0.0001 which are different from the value of control. ^*ƚ*^ *P* < 0.0001 which is different from the value of 0.1% GE. ^a^ *P* < 0.001, ^b^ *P* < 0.0001 which are different from the value of 0.5% GE.

The demineralized dentin collagen treated with GE exhibited distinct features. Dentin collagen in the control group presented a white color while a concentration- and time-dependent dark blue pigmentation was observed in the GE-treated groups (Figure 1). Hyp analysis showed that dentin collagen in all 18 control groups was almost completely digested. However, when treated with GE, the digestibility markedly decreased in a concentration- and time-dependent manner (Figure 2). The Hyp contents in the insoluble residues (nM ± SD, treatment time in parenthesis) in the samples treated with 0.5% GE were 81.9 ± 4.9 nM* (30 min), 166.4 ± 3.8 nM* (1 h), 926.6 ± 37.6 nM** (4 h), 1,243.5 ± 50.6 nM** (8 h), 1,259.1 ± 48.3 nM** (12 h), and 1,259.3 ± 61.4 nM** (24 h). Twenty-four-hour treatment with PBS (control), 0.01% GE, and 0.1% GE resulted in the recovery of 2.0 ± 1.7 nM^b^, 166.0 ± 19.3 nM^∗,b,*ƚ*^, and 1,054.3 ± 47.3 nM^∗∗,a^ Hyp in the insoluble residues, respectively (*P < 0.001, **P < 0.0001 which are different from the value of control. ^*ƚ*^ *P* < 0.0001 which is different from the value of 0.1% GE. ^a^ *P* < 0.001, ^b^ *P* < 0.0001 which are different from the value of 0.5% GE) (see Figure 2).

### 3.2. Amino Acid Analysis

Amino acid compositions in all groups at all-time points were essentially identical to one another (data not shown) with the exception of Lys and Hyl. The amounts of Lys and Hyl in all groups treated for 24 h are shown in Table 1. Lys and Hyl in the GE-treated groups were significantly decreased in a concentration-dependent manner when compared to the control. No significant changes were observed for other amino acids.

### 3.3. Collagen Cross-Link Analysis

**Figure 3:**
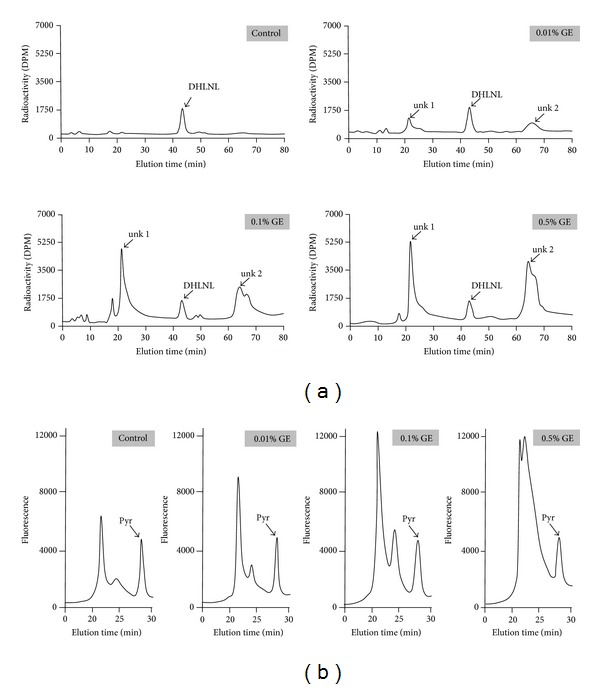
Representative chromatographs of (a) reducible and (b) nonreducible collagen cross-links in 24 h treatment duration of PBS (control) and three concentrations of genipin (GE) (0.01%, 0.1%, and 0.5%) groups.

Typical chromatographic profiles of reducible and nonreducible collagen cross-links are shown in Figures 3(a) and 3(b), respectively. Two major cross-links, DHLNL (reducible) and Pyr (nonreducible), were identified in all groups. In all of these samples, HLNL and d-Pyr were less than 1/10 of DHLNL and Pyr, respectively, and thus they were not calculated. In addition, two unknown (unk), NaB^3^H_4_-reducible peaks were identified in the GE-treated groups (peaks at 22 min: unk 1, and peaks at 64 min: unk 2, resp.) (Figure 3(a)). The results of the quantitative cross-link analyses of DHLNL, Pyr, unk 1, and unk 2 comparing PBS- and GE-treated groups are summarized in Table 2.

The amounts of DHLNL in 0.01% and 0.1% GE for 24 h were not significantly different from those of control, but DHLNL in 0.5% GE for 24 h (1.13 ± 0.07 M) was significantly decreased when compared to that of control (1.36 ± 0.17 M) (*P* < 0.05) (Figure 3(a) and Table 2). The amount of Pyr in all three GE groups treated for 24 h showed no significant differences when compared with control (Figure 3(b) and Table 2).

The amounts of newly GE-induced reducible cross-links/compounds, unk 1 and unk 2, in GE groups treated for 24 h were significantly increased in a concentration-dependent manner (Figure 3(a) and Table 2).

## 4. Discussion

The present study was undertaken to evaluate the effects of GE treatments under various conditions on dentin collagen using biochemical approaches such as Hyp analysis, amino acid analysis, and quantitative cross-link analysis. GE-treatment of collagen resulted in blue pigmentation as previously reported [24, 30, 40]. The blue discoloration of demineralized dentin collagen by GE increases in a concentration- and time-dependent manner. The blue pigmentation is possibly formed through a series of oxygen radical-involved polymerization and dehydrogenation of several intermediary pigments [41, 42] utilizing the *ε*-amino group of Lys and Hyl. These compounds are likely associated with intra- and intermolecular cross-linking of collagen as the intensity of the color corresponded well with the collagen stability against enzymatic digestion. Collagen stability was evaluated by digestibility with bacterial collagenase which hydrolyzes the peptide bond on the amino-terminal side of Gly in -X-Gly-Pro [43]. Based on the mean values of Hyp in 2 mg demineralized dentin matrix in this study, the rate of collagen digestion was 93.6%, 86.9%, 27.2%, 2.3%, 1.1%, and 1.1% in 0.5% GE for 30 min, 1 h, 4 h, 8 h, 12 h, and 24 h, respectively. Treatment with PBS, 0.01% GE, and 0.1% GE for 24 h resulted in 99.8%, 87.0%, and 17.2% collagen digestion, respectively. Thus, the rate of collagen digestion with GE treatment clearly decreased in a concentration- and time-dependent manner (Figure 2). Almost no collagen was digested when collagen was treated with 0.5% GE for 12 h and 24 h. We hypothesize that GE-induced cross-linking hinders the enzyme accessibility to collagen and/or generates a large cross-linked collagen complex so that collagenase cleavage no longer solubilizes the complex. Further studies are warranted to test this hypothesis.

Results of the amino acid analysis demonstrated that Lys and Hyl residues were the only amino acids that decreased significantly with GE treatment in a concentration-dependent manner. When the values of Lys and Hyl were calculated on residues per 1,000 amino acids basis, the mean number of Lys in GE groups for 24 h decreased from 26.2 (±3.5) in the control group to 2.3 (±1.6) in the 0.5% GE group. In the case of Hyl, it decreased from 12.8 (±0.8) in the control group to 1.6 (±0.4) in the 0.5% GE group (note: some of the Lys residues are derived from noncollagenous proteins). These data indicate that approximately 90% of both Lys and Hyl residues were utilized for GE-induced cross-links when treated with 0.5% GE treatment for 24 h. Since some of the Hyl residues in dentin type I collagen are glycosylated [44], the data also indicate that the posttranslational modifications of Lys, that is, 5-hydroxylation, and subsequent O-glycosylation do not significantly hinder the formation of such cross-linking. Under the conditions used, no other amino acids including arginine (Arg) were significantly changed by GE treatment. Sung et al. reported that Arg in addition to Lys and Hyl was used in the reaction with GE in porcine pericardia [24]. Possibly, under the treatment conditions and tissues used in the current study, the GE reaction favors the *ε*-amino groups on Lys and Hyl.

Cross-link analysis revealed at least two unidentified, reducible compounds in the GE-treated groups. These major radioactive peaks eluted at 22 and 64 min were observed in our HPLC system, and they increased in a concentration-dependent manner. Thus, these two newly formed reducible compounds are likely associated with GE-induced cross-links involving Lys and Hyl residues of collagen. Two cross-linking mechanisms between GE and biopolymers (such as collagen) containing primary amine groups were proposed by Butler et al. [45], one via the nucleophilic attack of the GE C3 atom from a primary amine group forming an intermediate aldehyde leading to the formation of heterocyclic compound linking GE and the biopolymer and the other via nucleophilic substitution of the ester group in GE forming a secondary amide link to the biopolymer. However, a number of intermediates [41, 42] and further condensations/polymerization [46] are likely to occur. Some of these cross-linking compounds could be reduced with NaBH_4_ and stabilized. Further studies are warranted to identify the structures of these cross-links by isolating and characterizing these compounds and collagen-derived cross-linked peptides by, for instance, biochemical and mass spectrometric approaches [13]. There was a slight but significant decrease of DHLNL in 0.5% GE group treated for 24 h when compared to that of control. The cause of this decrease is not clear but it could be due to the GE-induced modification of the unstable aldimine bond of deH-DHLNL which was present prior to its rearrangement to a stable ketoamine. The decrease in DHLNL, however, needs to be confirmed by increasing the number of analyses. The maturational product of deH-DHLNL/its ketoamine, Pyr, showed no significant changes in all groups in this study due likely to the stability of this cross-link.

Further characterization of GE-induced cross-links in dentin collagen and their long-term stability, dentin mechanical properties, and restorative procedures is warranted. A natural compound-based dentin treatment for tooth or restoration reinforcement may help develop a reliable and safer therapy with applications in dentistry.

## 5. Conclusions

Under the conditions of this study, the treatment of bovine dentin collagen with GE results in a concentration- and time-dependent increase in discoloration and collagen stability against bacterial collagenase. The GE-induced, reducible cross-links/compounds involving Lys and Hyl residues increase in a concentration-dependent manner. GE treatment may modify some of the immature divalent cross-links.

## Figures and Tables

**Table 1:** The amounts of lysine and hydroxylysine in a 24-hour treatment duration of PBS (control) and three concentrations of genipin (0.01%, 0.1%, and 0.5%) groups.

	Control	0.01% GE	0.1% GE	0.5% GE
Lys	26.2 (3.5)	18.8 (0.8)^a^	9.8 (2.3)^c,e^	2.3 (1.6)^c,f,h^
Hyl	12.8 (0.8)	7.5 (1.8)^b^	4.3 (0.3)^c,d^	1.6 (0.4)^c,e,g^

All values are shown as relative amounts in 1,000 total residues (means and standard deviations). GE: genipin; Lys: lysine; Hyl: hydroxylysine; *n* = 3; ^a^ *P* < 0.05 which is different from the value of control; ^b^ *P* < 0.005 which is different from the value of control; ^c^ *P* < 0.001 which is different from the value of control; ^d^ *P* < 0.05 which is different from the value of 0.01% GE; ^e^ *P* < 0.005 which is different from the value of 0.01% GE; ^f^ *P* < 0.001 which is different from the value of 0.01% GE; ^g^ *P* < 0.05 which is different from the value of 0.1% GE; ^h^ *P* < 0.01 which is different from the value of 0.1% GE.

**Table 2:** The contents of enzymatic and GE-induced cross-links in a 24-hour treatment duration of PBS (control) and three concentrations of genipin (0.01%, 0.1%, and 0.5%) groups.

	Control	0.01% GE	0.1% GE	0.5% GE
DHLNL	1.36 (0.17)	1.35 (0.23)	1.23 (0.19)	1.13 (0.07)^a^
Pyr	0.190 (0.085)	0.188 (0.014)	0.187 (0.084)	0.186 (0.083)
unk 1 (22 min)	3169 (793)	26991 (4007)	110483 (6525)^a,f^	142893 (25133)^c,e^
unk 2 (64 min)	1787 (90)	29160 (8418)	136342 (21531)^b,d^	253668 (45338)^b,e^

All values in DHLNL and Pyr are expressed in moles/mole collagen (means and standard deviations). All values in unk 1 and 2 are expressed in disintegrations per minute (DPM) (means and standard deviations). DHLNL: dihydroxylysinonorleucine; Pyr: pyridinoline; unk: unknown; GE: genipin; *n* = 3; ^a^ *P* < 0.05 which is different from the value of control; ^b^ *P* < 0.01 which is different from the value of control; ^c^ *P* < 0.001 which is different from the value of control; ^d^ *P* < 0.05 which is different from the value of 0.01% GE; ^e^ *P* < 0.005 which is different from the value of 0.01% GE; ^f^ *P* < 0.001 which is different from the value of 0.01% GE.
